# Activity of Peptides Modulating the Action of p2x Receptors: Focus on the p2x7 Receptor

**DOI:** 10.3390/ph18101452

**Published:** 2025-09-28

**Authors:** Jonathas Albertino De Souza Oliveira Carneiro, Guilherme Pegas Teixeira, Leandro Rocha, Robson Xavier Faria

**Affiliations:** 1 Laboratory for Evaluation and Promotion of Evaluation and Promotion of Environmental Health (LAPSA), Oswaldo Cruz Institute, Oswaldo Cruz Foundation, Rio de Janeiro 21040-900, Brazil; universodigital.tj@gmail.com (J.A.D.S.O.C.); gpegas67@gmail.com (G.P.T.); 2 Postgraduate Program in Science and Biotechnology, Institute of Biology, Fluminense Federal University, Niterói 24210-130, Brazil; 3 Postgraduate Program in Vegetal Biotechnology and Bioprocesses, Rio de Janeiro Federal University, Rio de Janeiro 21941-902, Brazil; lean.machado@gmail.com; 4 Laboratory of Natural Products Technology, Faculty of Pharmacy, Fluminense Federal University, Rua Doutor Mário Viana 523, Santa Rosa, Niterói 24241-002, Brazil

**Keywords:** peptides, P2X receptors, P2X7 receptors, purinergic receptors

## Abstract

P2X receptors are a family of ATP-gated ion channels widely distributed in various tissues, especially in neuronal cells and hematopoietic cells. ATP activates P2X receptors, causing the opening of an ionic channel with preferential permeability to the passage of mono- and divalent cations. High concentrations of ATP stimulate the P2X7 subtype through prolonged activation, which opens pores and causes inflammation, proalgesic effects, and cell death. Peptides, including antimicrobials (antimicrobial peptides), are present in several organisms, such as amphibians, mammals, fish, arachnids, and plants, where they act as the first line of defense. Thus, these peptides have the capacity to eliminate a wide spectrum of microorganisms, such as bacteria, fungi, and some viruses. In general, the mechanism of action of antimicrobial peptides involves interactions with the lipid bilayer of the cell membrane, which can lead to an increase in the internal liquid content of liposomes. However, many peptides can act on ion channels, such as those of the P2X family, especially the P2X7 receptor. We investigated the action of peptides that directly modulate P2X7 receptors, such as beta-amyloid, LL-37/hCap18, Pep19-2.5, rCRAMP, ADESG, and polymyxin B. Additionally, we evaluated peptides that modulate the activity of P2X family receptor subtypes. In this review, we intend to describe the relationships between peptides with distinct characteristics and how they modulate the functionality of P2X receptors.

## 1. Introduction

P2X receptors are ionotropic and are activated by ATP. They form nonselective cation channels that allow the passage of Na^+^/K^+^/Ca^2+^ ions through a concentration gradient [1]. This family has seven members: P2X1, P2X2, P2X3, P2X4, P2X5, P2X6 and P2X7 in mammals. The influx of calcium and consequent depolarization of the membrane activate voltage-dependent calcium channels, which further increase the intracellular calcium concentration. When activated, P2X receptors increase the level of intracellular calcium (Ca^2+^) and modulate pathological processes such as pain, inflammation, and neuronal dysfunction through the activation of cellular signaling pathways, exocytosis, and even cell damage. Therefore, P2X receptors are promising therapeutic targets for preventing these detrimental effects and alleviating pain and inflammation in patients with diseases such as neuropathic pain and chronic kidney disease [1,2].

The development of therapies targeting P2X receptors has been a long and complex journey that began with basic research characterizing the receptors involved in health and disease, ultimately leading to the identification of potential therapeutic targets for various conditions. Although some P2X3 and P2X7 receptor antagonists have reached the clinical level, these drugs are not effective [3].

Peptides can vary in length and amino acid composition, and these elements determine their physicochemical properties and biological functions, including regulating hormonal activity, modulating immune responses, and influencing intestinal health. Among the different mechanisms of action [4], peptides can act as ligands for plasma membrane receptors.

The discovery of high-affinity peptides for receptor recognition holds strong potential for enabling new therapeutic agents, such as inhibitors/activity modulators [5].

Thus, we explored studies on peptides with modulatory effects on P2X receptors, evaluated their pharmacological and physicochemical elements and related them to functional aspects. In this way, we understand that the binding interactions controlling the recognition of peptide sequences are expected to pave the way toward the rational design of synthetic receptors for surface protein recognition.

## 2. Peptides

These molecules play crucial roles in various physiological processes, acting as messengers in cellular communication, modulators of the immune response, hormone regulators, and other functions [6].

Owing to their high specificity and biological activities, peptides are of great interest in pharmaceutical research and are the target of studies as potential bioactive pharmaceuticals in various therapies. Extracellular peptides are capable of modulating the activity of the P2X7 receptor and reducing inflammation [7,8]. Likewise, peptides derived from anti-inflammatory proteins, known for their ability to respond to inflammation, have also been investigated for their capacity to modulate the P2X7 receptor [9,10].

This statement is justified by previous studies investigating the inhibitory capacity of peptides, such as Hidrostatima-SN1 [11], cathelicidin LL-37 [12], Canus [13], and others, which can inhibit P2X7 receptor function [14].

## 3. Structure of P2X7

P2X receptors have a trimeric structure and are nonselective cation channels activated by extracellular ATP [15] and are considered promising pharmacological targets [16]. This is justified by the variety of eukaryotic cells that modulate key physiological mechanisms, such as platelet activation [17], synaptic action, muscle contraction, inflammatory processes, and apoptosis [18].

P2X receptor pharmacology is still incompletely understood at the molecular level. Previous studies have shown that the cytoplasmic termini of P2X receptors play a key role in receptor desensitization [19]. However, the molecular mechanism underlying desensitization remains poorly understood due to the lack of structural information [20]. Notably, the absence of ATP stimuli and the complete absence of desensitization, the P2X7 receptor is distinguished from other members of the P2X family, since P2X7 is the only subtype that acts as a receptor for multiple signaling pathways [21].

The gene encoding human P2X is located on chromosome 12 and is centromeric and proximal to P2X4. Thus, mouse P2X7 and P2X4 are located on chromosome 5. Thus, the proximity of P2X4 and P2X7, unified by sequence homology, predicts the functional value of the substituted amino acids on the alignment of highly similar orthologous and paralogous protein sequences, suggesting an origin by gene duplication [22,23].

The P2RX7 receptor encodes a 595-aa protein (the P2X7 subunit or monomer) that assembles into a trimeric complex to generate the functional P2X7R. The P2X7 receptor monomer contains a short intracellular residue of 26 aa, a bulky extracellular domain of 282 aa, two transmembrane helices of approximately 24 aa each, and a long carboxy-terminal cytoplasmic tail of 239 aa [24]. The most interesting domain in the P2X7 receptor subunit is the carboxy-terminal tail, comprising amino acids 356–595, which is specific to this receptor subtype [25,26].

**Figure 1:**
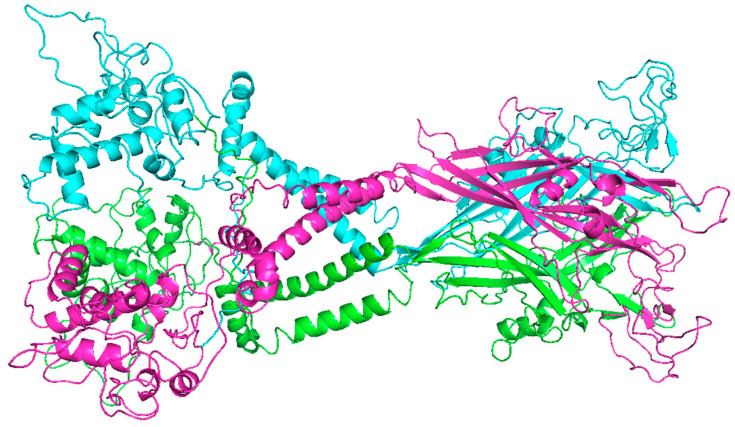
Image created by the author via PyMOL software (The PyMOL Molecular Graphics System, Version 3.0 Schrödinger, LLC.) Representing the structure of the P2X7R receptor as a rat ATP-gated ion channel in the closed state. This receptor has 609 amino acids and a theoretical weight of 69.9 kDa (the source organism is *Rattus norvegicus*, expressed by *Homo sapiens*). This image represents its current representative figure in structural format by cryo-electron microscopy of the P2X7 receptor ion channel, accessing the PDB server and the European Protein Data Bank with the international protein code 6U9V at https://www.ebi.ac.uk. (accessed on 15 May 2025).

To better understand the structure of the P2X7 receptor, single-particle cryo-electron microscopy was used to determine the structures of P2X7 from a mammalian organism, specifically a rat, in a closed pore state linked to ATP (open pore). This cryo-electron microscopy-based investigation builds upon previous studies, which have suggested that the P2X7 receptor subtype has a low affinity for ATP, with approximate EC50 values of 100–300 µM [15](Figure 1).

## 4. P2X7 Receptor Function

The P2X7 receptor is stimulated by extracellular adenosine 5′-triphosphate and induces a series of responses in the body and a proinflammatory response [15]. Therefore, the P2X7 receptor has emerged as an excellent target for the development of therapies, particularly those related to inflammatory and neurodegenerative conditions.

This ionotropic receptor intervenes in the activation of the NLPR3 inflammasome, the release of cytokines and chemokines, the survival and differentiation of T lymphocytes, the activation of transcription factors, and cell death. In terms of inflammatory activities and immunity, the P2X7R receptor affects (cellular lymphocytes and macrophages) and pharmacological (chemokines and cytokines) responses [27].

Recent studies revealed that synthetic compounds derived from plant act on the P2X7 receptor and modulate its function, subsequently reducing inflammatory and alginic responses in vitro [28] and in vivo [29]. In this review, several peptides that have been discovered over the years as potential inhibitors of P2X7R have been developed and studied, and their mechanism of P2X receptor inhibition has been explored [29].

## 5. Peptides with Modulatory Activity Against the P2X7 Receptor

Peptides can modulate the immune response and interact with specific receptors, such as P2X7, which has potential as a therapeutic target for the treatment of a wide range of inflammatory diseases [30]. We highlighted several peptides with modulatory effects on the P2X7 receptor Table 1 [30,31].

### 5.1. Beta-Amyloid Peptide

Alzheimer’s disease is a neurodegenerative disease that affects the central nervous system. Several studies have shown that aging is the primary risk factor for the development of this disease. However, previous research has also revealed other factors contributing to its development [31]. The presence of senile plaques in the brain acts as a marker for Alzheimer’s disease. However, the presence of amyloid plaques is necessary but not sufficient to obtain a diagnosis of Alzheimer’s disease [41].

The amyloid beta peptide contains a complex of 42 amino acids generated from the amyloid beta protein. This protein is classified as a transmembrane glycoprotein that crosses the membrane once [42]. The ability of extracellular ATP to act on P2X7Rs plays a crucial role in the neuroinflammation and neurotoxicity induced by the amyloid beta peptide (Aβ). The P2X7R receptor is upregulated in the microglia surrounding senile plaques, and this upregulation progressively increases with age; the older the individual is, the more progressive the Alzheimer’s pathology is [43]. The early symptoms associated with Alzheimer’s disease suggest that changes associated with aging may be relevant in the progression of the disease.

The number of microglia, together with their ability to respond to exogenous ATP stimulation, decreases with age. Thus, age-related changes in P2X7R in microglia may be decisive for the progression of this disease [44]. Therefore, in experimental tests, a transgenic mouse model created from the crossing of a mouse with beta-amyloid peptide (J20 mouse) and P2X7R reporter mice indicated that the inflammatory process induced by this peptide caused changes in the distribution pattern of the P2X7R receptor, increasing its expression in microglia in advanced stages of the disease [42]. This study revealed that pharmacological inhibition or selective downregulation of the P2X7R receptor improved behavioral changes, reducing the incidence and size of senile plaques in early and advanced stages of Alzheimer’s disease, thus providing evidence that this peptide may be a therapeutic tool for Alzheimer’s disease treatment [42,43,44,45].

As in other studies supporting this argument, amyloid protein (A-) increased the level of intracellular calcium and promoted ATP release, IL-1 secretion, and plasma membrane permeabilization in normal microglia. However, these effects were not observed in microglia from mice lacking the P2X7 receptor. Similarly, the injection of amyloid into the hippocampus of wild-type mice resulted in a large accumulation of IL-1, which did not occur in mice lacking the P2X7 receptor. These findings suggest that beta-amyloid peptide activates a purinergic stimulation cycle (involving ATP) in which the P2X7 receptor is an essential component. The identification of the P2X7 receptor as a crucial factor in amyloid-mediated microglial activation may therefore open new possibilities for the treatment of Alzheimer’s disease [46].

### 5.2. LL-37/hCap18 Peptide

The human cathelicidin LL-37 plays a critical role in the innate immune system, acting to combat bacterial infections. Currently, we are facing a significant increase in antibiotic resistance, which is becoming a major challenge to global health. Given this problem, studies highlight the importance of advancing research that addresses the discovery of new bactericidal drugs, including antimicrobial peptides, owing to their potential, on the basis of their main structures, to eradicate pathogenic bacteria [47]. However, many antimicrobial peptides, in contrast to a small portion of these antimicrobial peptides, have been introduced in clinical studies, and none have entered the pharmaceutical market [47].

Therefore, understanding the mechanisms by which antimicrobial peptides target and kill bacterial cells to create peptide formulators with specific properties to combat bacterial infections is important [47]. Current studies indicate that the bioactive peptide LL-37/hCAP18, as an integral part and the only human member of the cathelicidin family, plays a crucial role in the elimination of various pathogens, as well as in immune modulation [48]. The P2X7R receptor plays an important role in the internalization of LL-37 by human macrophages. LL-37 was detected in macrophages pretreated with P2X7R antagonists and in differentiated THP-1 cells in which the P2X7R gene had been silenced [48]. Therefore, the internalization of LL-37, which is mediated by P2X7R, is linked primarily to a clathrin-mediated endocytosis pathway. These findings demonstrate that internalized LL-37 traffics to endosomes and lysosomes, contributing to the intracellular clearance of bacteria by human macrophages. These human macrophages have the potential to import LL-37, a peptide released from activated human neutrophils, revealing a novel mechanism by which these human macrophages internalize antimicrobial peptides to increase the clearance of intracellular pathogens [48]. The results revealed that human macrophages internalize the antimicrobial peptide LL-37 through a P2X7R receptor-dependent mechanism. A clathrin-dependent pathway was involved, leading to increased pathogen clearance. Thus, LL-37 and other antimicrobial peptides may be promising molecules for combating the inflammatory process and infections, suggesting that LL-37 could be a potential treatment in the future.

Additionally, another study reinforced this statement, highlighting that extracellular ATP and the LL-37 peptide activate the P2X7 receptor, a fundamental process in inflammatory responses and tissue damage. LL-37 promotes cell proliferation, calcium (Ca^2+^) influx, and iodide (I^−^) uptake in combination with benzoyl ATP to optimize the functions of P2X7 pores and channels. This activity of LL-37 is completely dependent on P2X7 expression and is influenced by the structural properties of LL-37, such as its ability to form helices. Interestingly, LL-37 can induce pore formation even with a truncated form of the P2X7 receptor, which indicates that P2X7 is crucial in the cell proliferative response to LL-37 and that the structural characteristics of LL-37 modulate the activation of this receptor [35].

### 5.3. Peptide Pep19-2.5

The synthetic LPS-neutralizing peptide Pep19-2.5 was investigated for its biological activity toward human P2X receptors in innate immune cells. The effect of Pep19-2.5 on P2X7R partially affects the influx of Ca^2+^ in addition to the release of interleukins and lactate dehydrogenase (LDH). Confocal microscopy was used to confirm the localization of Pep19-2.5 at P2X7R receptors. In the presence of the P2X7R receptor, the Pep19-2.5 peptide reduced the release of IL-1 and LDH with an IC_50_ of 0.346 μM in 1321N1 astrocytoma cells stably transfected with P2X7R, demonstrating that this potential LPS-neutralizing peptide acts as a P2X7R receptor modulator [36].

Another group presented a hypothesis about the involvement of the P2X7R receptor and the Pep19-2.5 peptide, through which Ca^2+^ influx was analyzed in the presence of the P2X receptor inhibitor pyridoxalphosphate-6-azophenyl-2,4-disulfobonic acid (PPADS). A complete blockade of Ca^2+^-induced Pep19-2.5 in the presence of PPADS occurred, and this process was, at least partially, dependent on the participation of P2X7R. HEK293T cells transfected with P2X7R and treated with the Pep19-2.5 peptide presented a sustained increase in intracellular Ca^2+^ only in cells expressing the human P2X7R receptor, like BzATP. Therefore, Pep19-2.5 acts directly on the inflammatory process mediated by P2X7R [36].

### 5.4. rCRAMP Peptide

This antibacterial peptide, rCRAMP, which is homologous to human LL-37, has demonstrated not only potent bactericidal activities but also functions as a chemoattractant for immune cells. A previous study investigated the role of this peptide in the innate immunity of brain cells and demonstrated the impact of rCRAMP on the activation of glial cells [37]. In the present study, the cathelicidin rCRAMP, an antibacterial peptide, demonstrated not only strong bactericidal action but also the ability to attract immune cells in rats. Therefore, this study identified the CRAMP peptide as a stimulator of IL-6 production and ERK1/2 phosphorylation in glial cells. [36,37]

In another study, they reported that rCRAMP-induced signaling activated cytokine expression and regulated antimicrobial peptide levels in primary rat glial cells. Additionally, this study demonstrated the induction of proinflammatory cytokines and neurotrophic factors, as well as the activation of several signal transduction pathways, by rCRAMP in glial cells, as described previously [38,49]. This finding elucidates the efficacy of this peptide on the P2X7R receptor.

### 5.5. ADESG Peptide

The ADSEG peptide was investigated in a previous study in which a region called ADSEG, which is conserved among P2XR subtypes, was analyzed. This peptide was found in the M2 hydrophobic domain region of P2X7R and aligns with an H5 signature sequence of potassium channels [39].

The choice of this peptide was based on multiple sequence alignments via ClustalX software, which identified residues with 100% identity among all P2X7 receptor subtypes. This peptide exhibits relevant functionality, as the authors describe the TM2 segment as crucial for the formation of both a cationic pore and a nonselective pore, both of which are associated with P2X7R. The channel activity of the peptide has been demonstrated via artificial bilayers and biological membranes. The characteristics of this channel are remarkably similar to those of the full channel: the K^+^ conductance is 8.08 ± 0.08 pS under patch-clamp conditions and 8.0 ± 0.3 pS for P2X7R. The channel opening probabilities (K+) are 0.22 for lipid bilayers and 0.24 for the membrane patch-clamp, which are comparable to those of P2X7R (0.26) [39].

The study described here elucidates that a conserved and hydrophobic portion of the M2 segment can form ion channels in lipid bilayers, with properties that mimic those of the full P2X7 channel. These findings suggest that further research is needed to identify the specific residues involved in pore opening and to understand the mechanism of nonselective pore dilation. Additionally, the authors determined that there is an opening probability of ADSEG similar to that of the P2X7R receptor [39].

### 5.6. Polymyxin B Peptide

This peptide, known as polymyxin B (PMB), is an antibiotic that binds to and neutralizes bacterial endotoxin (LPS). The authors investigated the effects of this peptide on mouse- and human-mediated responses to explore the effects of P2X7R-related receptor agonists in HEK293 and K562 cells. Previous studies have investigated the effects of B lymphocytes in patients diagnosed with chronic lymphocytic leukemia. Thus, the PMB peptide significantly potentiated the effect of nucleotide ATP-mediated P2X7R stimulation. In contrast, in the presence of the polymyxin B peptide, the cells were killed by inefficient nucleotide concentrations, resulting in increased cytotoxicity, ATP-mediated Ca^2+^ influx, and plasma membrane permeabilization. Therefore, cells lacking the P2X7R receptor were completely insensitive to combined stimulation with polymyxin B and ATP, and polymyxin B at the concentrations used had no adverse effects on cell viability. These results demonstrate that PMB modulates the P2X7R receptor and suggest the need for in-depth and careful analysis when evaluating the responses of immune cells stimulated by ATP in the presence of polymyxin B peptide, which may not be affected by the removal of contaminating LPS alone [33].

In another study, polymyxin B (PMB) protected against LPS-mediated endotoxic shock in animals. The authors investigated how PMB affects P2X7 receptor responses in HEK293 and K562 cells (transfected with P2X7), mouse and human macrophages, and B lymphocytes from patients with chronic lymphocytic leukemia, aiming to explore P2X7 agonists in antitumor therapy. The authors observed PMB at a cell type-specific concentration, which dramatically potentiated P2X7 stimulation by nucleotides. Notably, ATP-induced Ca^2+^ influx, plasma membrane permeabilization, and cytotoxicity increased to the point that ineffective nucleotide concentrations became lethal in the presence of PMB. This synergistic effect between ATP and PMB was prevented by an irreversible P2X blocker (oATP) but not by a reversible antagonist (KN-62). Furthermore, cells that did not express P2X7 were immune to the combined stimulation, and PMB, at the concentrations used, had no negative effect on cell viability. These findings indicate that PMB is a valuable tool for modulating P2X7 function. These studies also suggest that ATP-stimulated immune cell responses in the presence of PMB should be evaluated with caution, as the observed effects may go beyond the simple removal of contaminating LPS [34,40]. Summary data on the peptides that modulate the P2X7 receptor are shown in Table 1.

## 6. Peptides Modulate the Activity of Other P2X Receptors

The action of copper can reduce the activity of ATP in P2X receptors. The carnosine dipeptide has been shown to selectively form Cu(II) complexes in vitro, indirectly enhancing ATP-evoked current signaling in P2X4 [50]. Similarly, previous work has shown that fragments of human octa-repeat prions (PrP60-67 or PrP59-9) prevent and reverse the 5 µM copper-induced ATP-evoked current, with EC_50_ values of 4.6 ± 1 and 1.3 ± 0.4 µM, respectively. In contrast, carnosine is less potent, with an EC_50_ of 44.4 ± 5.9 µM, in *Xenopus laevis* oocytes. These authors concluded that these findings lead to the formation of PrP–Cu(II) coordination complexes, which consequently reduce the modulation of this ion in P2X4 but do not involve direct binding to the receptor [51].

In 2019, a study involving a high-throughput fluorescence-based screening technique analyzed nearly 180 crude venoms from arachnids, centipedes, Hymenoptera, and cone snails for possible modulatory effects on P2X receptors. The main highlight of this work was the human P2X4 receptor, which exhibited mainly inhibitory activity in response to spider venom, as indicated by the quantification of YOPRO-1 and Fura-2 uptake. However, most substances responsible for this effect remain inconclusive [52].

The main peptide present in bee venom, melittin, is responsible for promoting hyperalgesia in a nonselective way. The “nonselective” context encompasses activity at transient receptor potential (TRP) channels, intracellular proteins, and phospholipase A2 [53]. Furthermore, a study by Lu and colleagues in which *Sprague Dawley* rats were stimulated with nociception and hypersensitivity induced by subcutaneous injection of 0.05 mg/50 μL melittin revealed that nociceptive effects were reversed with the use of 0.5 mg/20 μL A-317491 (a potent antagonist of P2X3 and heterooligomeric P2X2/3 receptors) in the peripheral region. These effects suggest that these receptors are involved in the hyperalgesic mechanisms of melittin [54].

BomoTx is closely related to a group of myotoxins, Lys49, a phospholipase A2-like protein extracted from *Bothrops moojeni* that excites a cohort of sensory neurons via ATP release and consequent activation of P2X2 and/or P2X3, two receptors widely expressed in these cells [55]. However, tests on HEK-293 cells transfected with P2X2 and P2X3 did not evoke currents after application of the toxin, suggesting that the action on these receptors would occur indirectly. The authors suggested that such ATP release was a result of the possible activity of hemichannels, such as pannexin and connexins, when 10 µM carbenoxolone (an antagonist of these channels) was used, which decreased ATP release [1]. At the experimental level in mice, 50 µM/20 µL BomoTx promoted nonneurogenic inflammatory pain, thermal hyperalgesia, and mechanical allodynia, the latter being dependent on purinergic signaling (as observed in P2X2/P2X3 knockout mice). Therefore, BomoTx may modulate ATP release via hemichannels, leading to the activation of P2X2 and P2X3 receptors, which in turn modulate mechanical hyperalgesia activity. TRPV1 has been proposed to promote nonneurogenic inflammatory pain and thermal hyperalgesia [56]. Another study using the Conus snail toxin Ω-conotoxin GVIA potently inhibited P2X3 and heterooligomeric P2X2/3 currents in rat DRG neurons, with IC_50_ values of 21.2 ± 1.7 nM and 3.84 ± 0.43 μM, respectively [57].

Savchenko and colleagues studied the activity of the constituents of *Geolycosa* spider venom, in which at least 7 proteins modulate various ionic currents via P2X receptors in murine sensory neurons [58]. Further investigation led to the isolation of a purotoxin-1 peptide that potently inhibits the P2X3 receptor with an IC_50_ of 12 nM in DRG neurons, which is 3-fold lower than that of the P2X3 and P2X2/3 receptor antagonist A-317491. Furthermore, intraplantar administration of 0.5 nmol significantly reduced the thermal hyperalgesia induced by carrageenan and CFA and the nociceptive behavior induced by capsaicin and formalin [59]. Purotoxin-2, another substance isolated from the same venom sample, also inhibited P2X3 expression in CHO cells. At a concentration of 50 nM, purotoxin-1 and 2 caused a 3- to 4-fold decrease in the IC_50_ of ambient ATP. Both toxins have modulatory effects on P2X3 without generating effects on oligomeric mP2X2/3 receptors [60].

Spinorphin is a neuropeptide from the bovine spinal cord that is an endogenous inhibitor of enzymes that degrade enkephalin, which is known for its analgesic activity [61]. Interestingly, the administration of 3 and 10 nM spinorphin strongly reduced the effects of 2-MeS ATP in male ddY mice [61]. Further investigation demonstrated that the tension clamp assay with recombinant human P2X3 receptors expressed in Xenopus oocytes was potent and noncompetitive concerning the human P2X3 receptor activated by ATP, with an IC_50_ of 8.3 pM, a value with high inhibitory potential [62].

Brain natriuretic peptide (BNP) is a natriuretic peptide hormone with significant potential for the treatment of cardiovascular diseases [63]. Interestingly, a study by Marchenkova et al. revealed that BNP can lead to the downregulation of P2X3 in the mouse trigeminal ganglion. Mechanistic investigations revealed two mechanisms underlying this effect: P2X3 serine phosphorylation and redistribution of the receptor to nonraft membrane compartments, which occur after BNP acts on the natriuretic peptide-A (NPR-A) receptor. Thus, the BNP-NPR-A pathway leads to the inhibition of P2X3 intracellularly [64].

Alpha hemolysin (HlyA) is a hemolytic protein and virulence factor secreted by uropathogenic strains of *Escherichia coli*. HlyA requires activation before being excreted into the extracellular environment and can modulate cells of the endothelium and the immune system [65]. As previously mentioned, studies have shown that ATP scavengers and nonselective inhibitors of P2X receptors (PPADS, BBG, and OxATP) inhibit HlyA-induced lysis in human, murine, and equine erythrocytes, suggesting the recruitment of P2X receptors. These authors highlighted the importance of P2X1 receptor antagonism, in which the selective inhibitor MRS2159 generated IC_50_ values of 150 and 250 μM for horse and mouse erythrocytes, respectively. In contrast, human erythrocytes were relatively insensitive to the antagonist but exhibited a small and statistically significant reduction at concentrations above 250 μM. A second selective P2X1 antagonist (NF449) inhibited HlyA-induced lysis in human erythrocytes in a dose-dependent manner, but it was less efficient in murine cells. Therefore, P2X1 receptor antagonism appears to play an important role in protecting against the hemolytic effects generated by HlyA [66].

Pep19-2.5 is a synthetic antimicrobial peptide with anti-inflammatory activity and good tolerability in mammalian cells [67]. Studies have reported that Pep19-2.5 can neutralize LPS-induced proinflammatory activity and activate or inhibit the P2X7 receptor [67]. Recently, the inhibitory potential of this peptide on other P2X subtypes was measured, revealing only an increase in intracellular Ca^2+^ in 1321N1 astrocytoma cells stably expressing human P2X1, P2X2, P2X3, and P2X4. P2X4 was the most notable, generating an IC_50_ of 0.146 μM, whereas P2X1 and P2X3 showed lower activities, with values of 4.23 and 10.1 μM, respectively. Only P2X2 was not promising, and its IC_50_ was above 10 µM. Taken together, these findings suggest that Pep19-2.5 interacts with P2X receptors with greater potency than P2X4 does [67]. Summary data on peptides that modulate other P2X receptors are shown in Table 2.

## 7. Conclusions and Perspective

Purinergic signaling, which is mediated by the P2X family of ATP-activated ion channels, results in a tangle of complex details with respect to pathophysiological processes, from difficult inflammatory processes to neurodegeneration. In short, the P2X7 subtype can transition to a permeable pore upon prolonged stimulation by high concentrations of ATP. In contrast, peptides recognized as having antimicrobial and immunomodulatory properties, such as the selective modulation of the functionality of P2X receptors, are intriguing. Thus, the relationship between peptides and P2X family receptors, especially the P2X7 receptor, orchestrates a molecular complex in which endogenous and exogenous peptides with specific structural and physicochemical characteristics exert various antagonistic effects on the potentiation of P2X7 receptor activity.

Therefore, the objective of this review was to evaluate the use of peptides that interact with P2X family receptors, especially the P2X7 receptor, a target protein for pharmacological studies on the production of drugs that combat the inflammatory process. Given the simplicity of P2X receptors as mere passive therapeutic targets, we instead suggest the important interactions that occur between peptides and P2X receptors, demonstrating that P2X receptors constitute a system of fine and dynamic regulation whose structures and functions still include further future analyses to develop new therapeutic strategies, with a deeper understanding of the associations of the various peptides already proposed in other works as new peptide designers. The idea of creating new natural or synthetic peptides that are efficient in combating the inflammatory process.

## Figures and Tables

**Table 1:** Functional characteristics of peptides and their effects on P2X7R [32].

Peptide	Target Receptor	Source Organism	Biological Effect	References
Beta-amyloid peptide	P2X7R	Amyloid precursor protein (APP)-Human	It causes an increase in intracellular Ca^2+^, ATP release, IL-1 release, and plasma membrane permeabilization in normal microglia.	[33,34]
LL-37/hCap18 peptide	P2X7R	The only cathelicidin present in humans	LL-37 stimulated cell proliferation, resulting in Ca^2+^ influx and promoting the activation of molecules capable of opening ion channels, such as benzoyl ATP, to optimize the functions of P2X7 receptor pores and channels.	[35]
Peptide Pep19-2.5	P2X7R	Synthetic antimicrobial peptide	Intracellular Ca^2+^ increase in 1321N1 astrocytoma cells stably expressing human receptor shows: IC_50_ of 0.346 μM for P2X7	[36]
rCRAMP peptide	P2X7R	Mouse cholin-related antimicrobial peptide	The rCRAMP peptide induced IL-6 expression and ERK1/2 phosphorylation in glial cells and in mice, inhibiting all responses coupled to the P2X7 receptor in macrophages.	[37,38]
ADESG peptide	P2X7R	Human protein, associated with the M2 domain of hP2X7R	HEK 293 cells were used in patch-clamp experiments in the cell- connected configuration.	[39]
Polymyxin B peptide	P2X7R	Mouse and human macrophages.	Human macrophages, HEK293 and K562 cells	[33,40]

**Table 2:** Functional characteristics of peptides and their effects on other P2X receptors.

Peptide	Target Receptor	Source Organism	Biological Effect	References
BomoTx- myotoxins Lys49	P2X2/P2X3	*Bothrops moojeni*	Indirect activation via ATP release by pannexin and connexin hemichannels in HEK-293 cells. Administration of 50 µM/20 µL of BomoTx promoted mechanical allodynia, and this effect was reversed with P2X2 and P2X3 knockout animals	[55]
BNP	P2X3	Mice	Downregulation of P2X3 in the mouse trigeminal ganglion by serine phosphorylation and distribution of the receptor to nonraft membrane compartments	[63]
Carnosine	P2X4	Human	Selectively form Cu (II) complexes with EC_50_ of 44.4 ± 5.9 µM in Xenopus laevis oocytes, increasing the current evoked by ATP	[50]
Human octarepeat prion PrP60-67	P2X4	Human	PrP-Cu (II) coordination complexes with EC_50_ of 4.6 ± 1 µM in Xenopus laevis oocytes, increasing the current evoked by ATP	[50]
Human octarepeat prion PrP59-9	P2X4	Human	PrP-Cu (II) coordination complexes with EC_50_ of 1.3 ± 0.4 µM in Xenopus laevis oocytes, increasing the current evoked by ATP	[50]
HlyA	P2X1	*Escherichia coli*	P2X1 antagonism blocks HlyA-induced erythrocyte lysis. MRS2159 generated IC_50_s of 150 and 250 μM for horse and mouse erythrocytes, respectively, whereas human erythrocytes showed a statistically significant reduction at a concentration above 250 μM	[65]
Melittin	P2X3/heterooligomeric P2X2/3	Bee venom	Subcutaneous injection of 0.05 mg/50 μL of melittin had nociceptive effects reversed with the use of 0.5 mg/20 μL of the antagonist A-317491	[53]
Ω-conotoxin GVIA	P2X3/heterooligomeric P2X2/3	Conus snail	Potently inhibited currents in rat DRG neurons with IC_50_ of 21.2 ± 1.7 nM and 3.84 ± 0.43 μM, respectively	[56]
Pep19-2.5	P2X1/P2X3/P2X4	Synthetic antimicrobial peptide	Intracellular Ca^2+^ increase in 1321N1 astrocytoma cells stably expressing human receptor shows: IC_50_ of 4.23 μM for P2X1 IC_50_ of 10.1 μM for P2X3 IC_50_ of 0.146 μM for P2X4	[36]
Purotoxin-1	P2X3	Geolycosa spider venom	IC_50_ of 12 nM in DRG neurons. Intraplantar administration of 0.5 nmol significantly reduced nociceptive behaviors	[58]
Purotoxin-2	P2X3	Geolycosa spider venom	CHO cells treated with 50 nM led to a 3- to 4-fold decrease in the IC_50_ dose of ambient ATP	[59]
Spinorphin	P2X3	Bovine spinal cord	IC_50_ of 8.3 pM in Xenopus oocytes expressing P2X3. Administration of 3 and 10 nM spinorphin strongly inhibited 2-MeS ATP activity in male ddY mice	[68,69]

## Data Availability

No new data were created or analyzed in this study. Data sharing is not applicable to this article.
